# Help! I Need a Remote Guide in My Mixed Reality Collaborative Environment

**DOI:** 10.3389/frobt.2019.00106

**Published:** 2019-11-15

**Authors:** Morgan Le Chénéchal, Thierry Duval, Valérie Gouranton, Jérôme Royan, Bruno Arnaldi

**Affiliations:** ^1^IRT b<>com, Rennes, France; ^2^IMT Atlantique, Brest, France; ^3^Lab-STICC, UMR CNRS 6285, Brest, France; ^4^Univ Rennes, INSA Rennes, Inria, CNRS, IRISA, Rennes, France

**Keywords:** 3D user interaction, collaborative virtual environments, virtual reality, augmented reality, mixed reality

## Abstract

The help of a remote expert in performing a maintenance task can be useful in many situations, and can save time as well as money. In this context, augmented reality (AR) technologies can improve remote guidance thanks to the direct overlay of 3D information onto the real world. Furthermore, virtual reality (VR) enables a remote expert to virtually share the place in which the physical maintenance is being carried out. In a traditional local collaboration, collaborators are face-to-face and are observing the same artifact, while being able to communicate verbally and use body language, such as gaze direction or facial expression. These interpersonal communication cues are usually limited in remote collaborative maintenance scenarios, in which the agent uses an AR setup while the remote expert uses VR. Providing users with adapted interaction and awareness features to compensate for the lack of essential communication signals is therefore a real challenge for remote MR collaboration. However, this context offers new opportunities for augmenting collaborative abilities, such as sharing an identical point of view, which is not possible in real life. Based on the current task of the maintenance procedure, such as navigation to the correct location or physical manipulation, the remote expert may choose to freely control his/her own viewpoint of the distant workspace, or instead may need to share the viewpoint of the agent in order to better understand the current situation. In this work, we first focus on the navigation task, which is essential to complete the diagnostic phase and to begin the maintenance task in the correct location. We then present a novel interaction paradigm, implemented in an early prototype, in which the guide can show the operator the manipulation gestures required to achieve a physical task that is necessary to perform the maintenance procedure. These concepts are evaluated, allowing us to provide guidelines for future systems targeting efficient remote collaboration in MR environments.

## 1. Introduction

**Figure 1:**
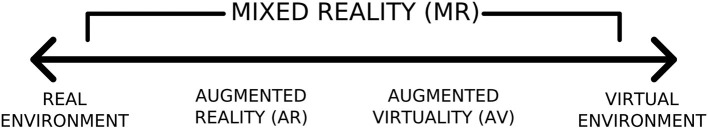
Milgram's continuum (Milgram and Kishino, 1994).

Mixed reality (MR) is a promising research area that combines the real world with virtual artifacts. It offers natural ways to display virtual content, taking advantage of real-world referencing in order to ease interactions. This leads to more immersive and intuitive systems, and thus improves user performance. According to Milgram's classification (cf. Figure 1), augmented reality (AR) overlays virtual objects into the real world, whereas augmented virtuality (AV) adds real items into a virtual environment (VE). The extremes of this classification are the real world and virtual reality (VR), i.e., a space that is purely virtual without the integration of real-world items.

Remote collaboration is a powerful tool in many situations, and can facilitate distant meetings, social communication, entertainment, teaching, etc. In particular, collaborative maintenance in which a remote expert helps an agent to perform a maintenance procedure has many advantages in terms of time and cost savings. However, many challenges are associated with remote collaboration, for instance:

- Perceiving a distant place in a comprehensive way;
- Being able to communicate using a shared referential system;
- Understanding a remote activity and another user's perception.

We can identify two research fields that involve work on these topics. Firstly, computer-supported cooperative work (CSCW) focuses on remote collaboration via a desktop computer. It mainly entails systems that can handle remote meetings and the concurrent editing of documents while maintaining consistency between distant physical locations (Rama and Bishop, 2006). Secondly, research on collaborative virtual environments (CVEs) takes advantage of VR technologies to offer remote collaboration in 3D VE, with more natural methods of perception and more intuitive interactions. These systems can provide shared 3D virtual worlds in which remote collaborators can interact via 3D virtual data and immersive telepresence systems that aim to decrease the real distance between users, allowing them to communicate as if they were in the same room (Zillner et al., 2014).

The remote guiding of an agent performing a physical task, helped by an expert, has already proved its usefulness in many proposed systems (Huang et al., 2013a; Robert et al., 2013). It can be used to achieve punctual unknown procedures, decreasing the time and the cost of interventions and allowing a single expert to remain in the same place while helping several potentially dispersed remote agents. However, one of the most difficult aspects of these systems is ensuring that collaborators understand what is happening in distant places. This is essential to be able to collaborate efficiently without misunderstanding. In this work, we propose innovative interaction techniques that focus specifically on this aspect of awareness in remote collaboration situations. In particular, our scientific contributions focus on evaluating these techniques in order to improve collaborative remote maintenance scenarios that can provide several advantages, for example:

- The agent can perform in the real world, with 1:1 scale interactions, and can be guided with an AR interface providing full perception of the real space;
- The remote expert can take advantage of a virtual interface to improve his or her perception of the distant space as well as his or her interaction capabilities to help the agent.

This leads to an imagining of different settings for remote expert interfaces and interactions. Firstly, we can provide a symmetric setting in which the remote expert can interact, as closely as possible, as if he or she were in the agent's workspace, on a 1:1 scale. Secondly, we can also imagine an asymmetric setting that provides a kind of world in miniature (WIM) view (Stoakley et al., 1995), allowing the expert a more global view of the shared scene, while the agent still interacts in the real world. Both of these scenarios have certain advantages depending on the current task, and can generate different user experiences and performances. In the same way, the agent's AR interface can be based on a head-worn or hand-worn display and cameras.

A head-mounted display (HMD)-based AR can intrinsically handle the co-location of this guidance, and thus avoids indirect mapping into the real world. A hand-worn display (HWD)-based AR does not directly display information into the real field of view of the agent, but this environment allows more freedom of motion of the camera used by the remote expert to perceive the state of the real workspace; however, it does not free the operator's hands, which imposes a constraint on the operator while performing the physical task.

This paper summarizes partially already published work by the current authors (Le Chénéchal et al., 2015, 2016) in section 4, as well as unpublished work in section 5. The aim of this is to unify our propositions into a comprehensive research approach, combining both symmetric and asymmetric approaches to build systems that best suit the needs of the users. In this paper, our main contribution is to demonstrate the advantages and drawbacks of our symmetric and asymmetric systems, based on several user studies. We show that our symmetrical approach can be very fast for rough guidance but could be improved in terms of precision, and that it limits unproductive movements of the operator's head. We also show that the proposed asymmetrical approach for remote guidance is very efficient in terms of speed and precision. Section 2 describes related work, and section 3 presents an overview of our proposals. Then, each approach is presented separately in sections 4 and 5. Lastly, we conclude this paper and discuss global results in section 6.

## 2. Collaborative Remote Guiding in Mixed Reality: The State of the Art

This research focuses on providing efficient interfaces that can enhance remote collaboration in MR. In particular, we limit our field of investigation to two remote collaborators and propose new ways to represent the distant workspace and the activities of the collaborators. In our scenarios, we divide the maintenance activity into two phases. Firstly, the expert must be able to diagnose the remote workspace to find how to perform the maintenance task. Secondly, the expert must be able to guide the operator to perform the actual physical task needed to achieve the maintenance procedure. Both of these phases require two kinds of interaction from the expert: navigation and manipulation. We can observe that navigation requires the expert to move remotely within the workspace via a shared VE built from sensors moved by the agent. Here, the difficulty lies not in controlling not a remote robot, but in guiding a human operator in handling sensors. In the following, we describe related work concerning collaborative navigation and manipulation guidance. Finally, we present work that focuses on enhancing awareness in a remote collaboration context in order to improve the usability and performance of the system.

### 2.1. Navigation

The issue of allowing a user to reach a specific viewpoint is not limited to CVE, and many single-user MR applications need to provide such a feature. Recently, Sukan et al. proposed the para-frustum approach to guide a user in an AR application to reach a set of acceptable viewpoints (Sukan et al., 2014). This paper presents an advanced technique based on a 3D augmentation that shows the user the viewpoint that should be reached. Compared to classical approaches, such as the use of a head-up display (HUD), this technique greatly eases the process due to the use of fully collocated guidance in the real interaction space. However, Sukan et al. highlight the complexity of defining the shape of the 3D augmentation, and it therefore seems complicated to extend it for collaborative purposes with definition of the shape carried out online by a distant user.

In CVE, we can distinguish between two approaches based on the system design: asynchronous and synchronous guidance. Asynchronous guidance is often used when a user defines several interesting viewpoints to another user, who can switch from one predefined viewpoint to another. In Duval et al. (2008), these interesting viewpoints are represented by virtual cameras surrounding a set of scientific data visualizations. Our proposals involve synchronous guidance. In this approach, a user interactively defines a viewpoint to be reached by another user. Many metaphors have been proposed (Nguyen et al., 2013), such as directional arrows, a compass, and a lit path. However, although these techniques work well in an asymmetric setup (i.e., when the guide has a global view of the scene while the visitor is immersed in it) they are difficult to extend to a symmetric, fully collocated setup. Indeed, even if their implementations can be fully automated (i.e., the guide does not need to define the parameters of the guidance cues, since the system is able to compute them automatically) they suffer from strict limitations, such as the loss of naturalness in the guidance process for the guided user due to the interpretation of specific visual cues and the eye strain that can be generated by the proximity of visual cues according to the guided viewpoint.

### 2.2. Manipulation

The literature presents remote collaborative systems based on shared wall-sized screens, allowing remote gesture guidance. A display can create a virtual window in the remote workspace, handling collocation with head-tracked stereoscopy (Towles et al., 2002), and even interaction with shared physical interfaces (Zillner et al., 2014). Most applications offering remote guidance for maintenance add interactive features, such as the ability to sketch guiding cues to help with navigation (Gauglitz et al., 2014a) with a correct 3D mapping onto the real world while the agent is moving, or a viewpoint-saving mechanism that facilitates the remote expert's task (Gauglitz et al., 2014b).

Alem et al. have developed several MR interfaces for remote collaborative maintenance based on a shared point of view. HandInAir (Huang et al., 2011) and MobileHelper (Robert et al., 2013) are examples of systems that provide gesture-based guiding for an agent helped by a remote expert. The agent wears a helmet with a camera that is streamed to the helper. The helper then carries out free mid-air gestures in front of a camera, and the helper's hands are merged with the output display of both users. Extensions using 3D capturing have been proposed, such as the HandsIn3D (Huang et al., 2013b) and 3D Helping Hands (Tecchia et al., 2012) systems. They include 3D handling of occlusions and virtual shadows, and thus improve immersion and the sense of presence. Nevertheless, the displays are still not collocated with the real environment, and the interaction of the helper remains limited to moving the head around the position of the agent's head and showing which gestures to perform, without being able to interact with any object. In the remainder of this paper, we aim to overcome these limitations by proposing techniques that can improve the available interactions for the expert and that decrease the perception issues experienced by both users, the expert and the operator.

### 2.3. Awareness

Awareness is defined as the internal/mental representation of a user according to the surrounding interactive world. This is a close definition of workspace awareness in a CSCW context given by Gutwin and Greenberg (2002). In VR, it is mainly achieved through the interpretation of sensory feedbacks that inform the user about the consequences of an action, and the self-updating of the VE. In CVE, this awareness of the shared VE is more complex, and remote collaborators must interpret what is happening in the shared VE, which is also a consequence of both users' actions. Thus, each collaborator's awareness involves the interpretation of signals sent by the other through the VE (Dourish and Bellotti, 1992). These signals can be handled via visual cues, such as gaze direction (Steptoe et al., 2008) or the facial expressions of avatars (Niewiadomski et al., 2011), or more coarsely via a field of view direction (Fraser et al., 1999). In addition, the states of interactive tools and objects can be represented to aid the user's own interactions and the understanding of remote interactions (Duval et al., 2013). Other communication channels can be used to improve remote collaboration. Verbal communication is the most natural choice, but video (Pauchet et al., 2007) and haptic (Chellali et al., 2010) channels can also be very helpful in some scenarios. Particularly in remote collaborative maintenance, this could offer a way to enhance perception of the remote workspace with tactile and force feedback from real furniture, in addition to its visual aspects. However, haptic devices are still expensive, not widely used and suffer from hardware limitations, such as a physical range of interaction; in our work, we therefore focus on visual feedback to enhance interactions with additional awareness cues.

This concept of awareness through visual cues has been well-explored in the literature, and some generic models have been proposed. For instance, the *focus/nimbus/aura* model (Benford and Fahlén, 1993) defines volumes attached to interactive entities. These volumes interact together according to their distance and handle awareness features regarding the properties and relations of entities. The IIVC model (Fleury et al., 2010), controls the awareness of interfaces (display, controllers, trackers, etc.) and associated constraints in order to decrease misunderstandings due to physical limitations in certain situations.

## 3. Overview of the Proposed Approaches

In this paper, we present our contributions in two distinct phases. Firstly, the remote expert must be able to diagnose the distant workspace and furniture while guiding the agent to follow him/her, in order to place the agent in the correct location. This navigation may take place in buildings containing corridors, rooms with complex shapes and furniture layouts, or with complex system shapes in industry. Thus, we propose new guiding techniques for the purposes of this navigation and evaluate them. Synchronous guiding techniques for remote navigation can be divided into two types, as follows:

- **Static guiding**: The expert points to a spot to reach, and the agent walks to this spot. This implies that the workspace perceived by the remote expert currently contains the desired spot (i.e., the expert can see the spot at the time that he/she offers guidance);
- **Dynamic guiding**: The expert continuously guides the agent to move in a certain direction, and can change the guiding direction at any time. This setting allows for dynamic discovery of the workspace by the remote expert, and makes it possible to guide the agent toward a spot that is initially not seen by the expert but is discovered dynamically due to the motion of the agent, who can move the streaming camera.

Once the desired spot for performing maintenance is reached, the physical manipulation guidance can begin.

### 3.1. Settings

In this work, we explore the following settings in order to manage collaborative remote maintenance:

- **(1) Symmetrical: Fully collocated**
  − The agent is guided via 3D collocated augmentations in the real field of view (using a see-though video HMD);
  − The expert is immersed in a 1:1-scale remote workspace (using an HMD) and guides the agent using real-scale interactions.
- **(2) Asymmetrical: HWD and WIM**
  − The agent is guided via a HWD;
  − The expert helps with a global view of the remote workspace.

The second setting uses simpler interfaces, and tends to be more deployable, while the first provides freehand interactions for the agent and immerses the remote expert in the maintenance workspace. However, both have advantages compared to existing solutions in the literature.

For navigation guidance, we propose both static and dynamic guiding conditions for setting (2), while for setting (1) the dynamic guiding condition is more relevant than the static one due to the 1:1 scale interactions of the expert, which are not designed to allow the pointing of a location that is out of reach to be seen.

For manipulation guidance, most systems propose visual guiding cues, such as sketches, notes, or arrows. However, these interaction techniques are not natural when guiding someone to perform a physical task. In real life guiding in a local context, the expert usually shows the operator how to proceed, at least once, and then the operator tries to reproduce this motion. Thus, with the aim of imitating this real life process, setting (2) makes it possible to display virtual clones of real objects that the expert can manipulate; these are integrated into the operator's real world due to the use of an AR display that manages occlusions and shadows to reduce depth cue conflicts, making it much less ambiguous to understand. Moreover, setting (1) allows the expert to make natural gestures at a 1:1 scale as guiding interfaces, either sharing the agent's viewpoint or in a face-to-face configuration, and this can also be more efficient than guiding via verbal instructions.

## 4. The Symmetrical Approach and Gestures-Based Guiding

In this section, we summarize our previous work on symmetrical systems (Le Chénéchal et al., 2015, 2016). Our fully collocated system aims to overcome the limitations encountered in remote maintenance applications, such as HWDs. Such systems must satisfy the following constraints:

- The agent must be able to perform free hand gestures, and to perceive augmentations integrated into the real space controlled by the expert, using an HMD;
- The remote guiding gestures must be easy to map onto the agent's real 3D world.
- The expert must be collocated in the distant workspace, in order to have the correct viewpoint of the workspace in terms of the scale of the scene, and to facilitate interaction;
- The expert must be able to interact with the virtual objects that are seen by the remote agent.

**Figure 2:**
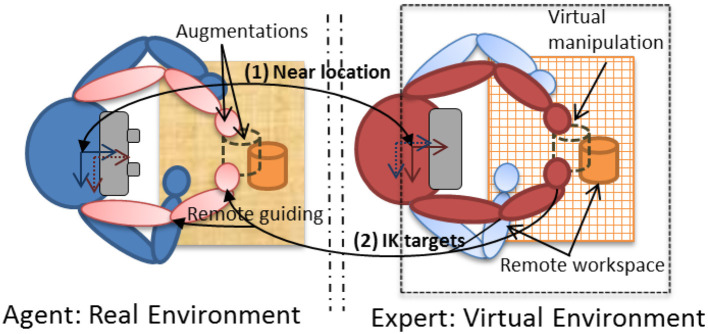
A conceptual top view of the *Vishnu* paradigm.

We therefore developed *Vishnu* (Le Chénéchal et al., 2016), a novel interaction paradigm that adds two virtual arms to a local agent, which are controlled by a remote expert. These two additional arms are overlaid from the agent's own shoulders, and the remote expert can use them as interaction tools to show the exact gestures and actions to perform, as illustrated in Figure 2. In this way, the interaction is more natural for the expert, and it is easier for the agent to understand the remote guiding instructions. Moreover, it provides the guide with the ability to share the exact PoV of the operator, which can improve his/her awareness of the operator's activity compared to an asymmetrical setting in terms of PoV, such as the HWD and WIM prototype presented in section 5 below.

### 4.1. System Design

**Figure 3:**
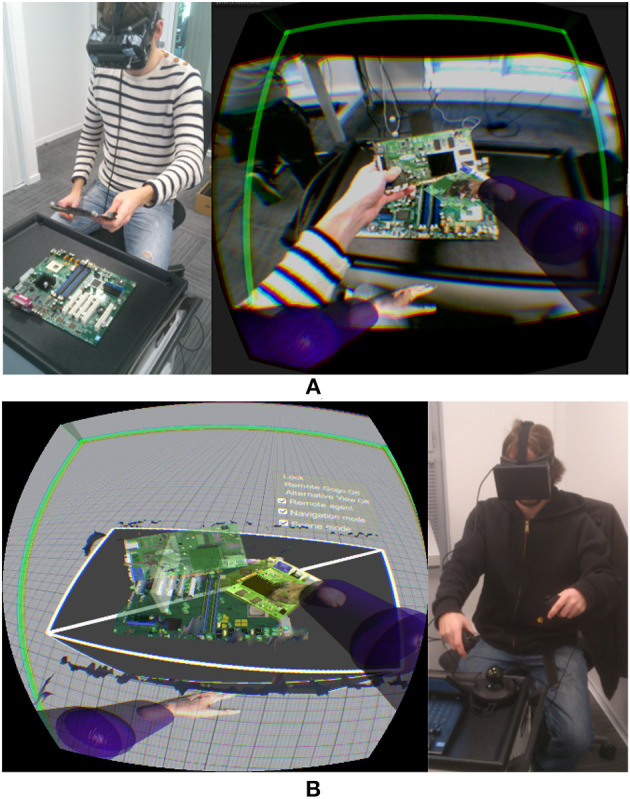
Illustration of the system and the viewpoints of **(A)** the agent and **(B)** the expert for a motherboard assembly task.

The main idea of *Vishnu* is to add two virtual arms to an agent, overlaid from the agent's own shoulders. Thus, the agent can see both his/her own real arms and the two additional virtual arms that are controlled by a remote expert, who can use them as interactive guidance tools. Figure 3 and additional Supplementary Video 2 illustrates this paradigm, which can be used in many applications, such as industrial maintenance, learning procedures and sports training. It is based on bi-directional communication (Figure 2), and the expert's virtual location must be close to the real position and orientation of the agent [step (1) in Figure 2]. The way in which the expert navigates within the shared VE is described in section 4.1.2. The virtual arm gestures of the expert define the inverse kinematics (IK) targets used to control the virtual arms of *Vishnu* for the agent [step (2) in Figure 2]. In *Vishnu*, the limitations mentioned above for both the expert and the agent are overcome, improving their sense of co-presence and decreasing their cognitive load, due to a de-localized display and interactions.

In the following, we present the features of our system, including the *Vishnu* paradigm and other components that combine to provide a highly usable system.

#### 4.1.1. Agent Features

A main goal of our system is to provide the agent with a true AR display, enabling proper perception of the augmented environment. To achieve this, we need to dynamically reconstruct the real environment in 3D in order to handle occlusions of real objects by virtual ones (see Le Chénéchal et al., 2016 for implementation details). This allows the handling of virtual shadows (i.e., virtual objects that are able to cast shadows on real objects), increasing the sense of presence and improving the perception of depth. The agent's head is also tracked to provide a collocated stereoscopic display.

The *Vishnu* paradigm is based on an IK algorithm (Tolani et al., 2000) that controls the virtual arms, with virtual targets for the elbows and hands, while the shoulders are fixed at the same location as the agent's. This is reasonable only if the agent is close to the expert's virtual location. Otherwise, the virtual targets are too far from the hands and elbows controlled by the expert, and the remote gestures are no longer relevant. In this system, the expert is represented with a viewing frustum that changes color from red to green according to the distance between the two users. This provides basic feedback to the agent, allowing him/her to find the correct placement when necessary. In the same way, the virtual arms smoothly disappear when the agent moves away from the expert's location.

#### 4.1.2. Expert Features

The most important limitation identified in previous work is the restricted interaction of the expert. Our system provides a VR setup for the expert that enables him/her to interact in the shared VE in a richer way. In addition to control over the viewpoint, the expert controls two virtual arms that can interact with virtual objects. Thus, the expert can grasp and manipulate virtual objects that are displayed as augmentations on the agent's side (Figure 3).

**Figure 4:**
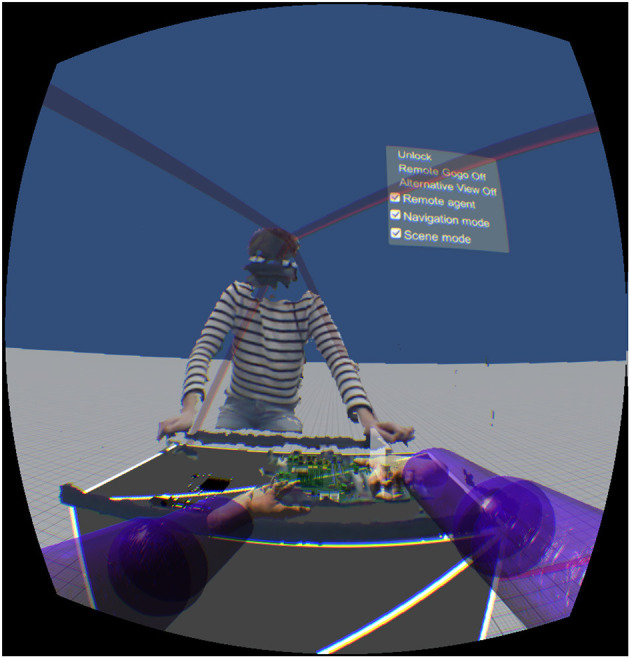
Illustration of the remote expert's viewpoint, in front of the distant agent (3D reconstruction).

The parts of the expert's arms (i.e., shoulders, elbows, and hands) are tracked and used to control their virtual representations to provide correct kinematics between the expert's gestures and the interactive tools. The manipulation of a virtual object is achieved using an interaction technique based on a virtual hand metaphor (Achibet et al., 2014). In *Vishnu*, the hands close around the virtual objects in a natural way using a simple grasping algorithm. Another interaction allows the expert to use the virtual hands to point to something. In order to point instead of grasping an object, the hand closes without the index finger, as illustrated in Figure 4.

In terms of navigation, the expert can freely move within the VE, but to guide the remote agent via gestures, the expert must be close to the agent's location. Thus, two interactions are possible:

- Smoothly returning the expert's location and orientation to the current position of the remote agent;
- Locking or unlocking the automatic following of the remote agent's head by the expert's virtual PoV.

When the expert shares the viewpoint of the agent, the navigation function is enabled. This allows the expert to guide the agent using the arms in Vishnu, in order to position the agent correctly to begin the manipulation task (see section 4.3).

Finally, a 3D reconstruction of the remote agent and workspace can be enabled, depending on current awareness needs. Particularly in the diagnostic phase, the expert may wish to stand facing the agent and freely explore the remote workspace, in order to find the correct location to perform the maintenance task (Figure 4).

### 4.2. Pilot User Study

**Figure 5:**
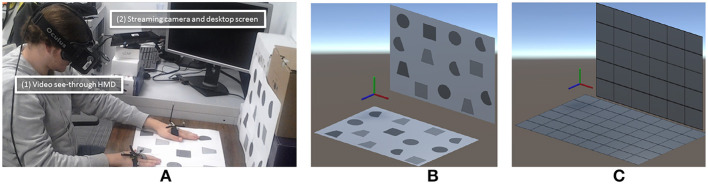
**(A)** A subject performing the pilot user study in mode 1 with the first target layout (L1). Notice the setup used for each mode: the transparent video HMD is used in mode 1, and in mode 2, the desktop screen on the side of the workspace is used to display sketches made by the expert, which are overlaid on the video stream of the camera. The virtual target spaces are reproduced from the real agent's workspace, as used in the expert's VE: **(B)**: first layout (L1); **(C)**: second layout (L2).

The details of the pilot user study are given in Le Chénéchal et al. (2015). Figure 5 illustrates our experimental setup, showing: (a) a subject performing the study, (b) the simple layout, and (c) the complex layout. This experiment allowed us to test the following hypotheses:

- H1: In a complex scenario, the subject's task completion, measured from the moment the guide finishes showing the task, is faster with our system (mode 1) than with a system involving a desktop screen on the agent's side and a fixed camera streaming video to the remote expert (mode 2).
- H2: In both the simple and complex scenarios, our system facilitates the mapping process between the guiding instructions and the physical task interaction space.

**Figure 6:**
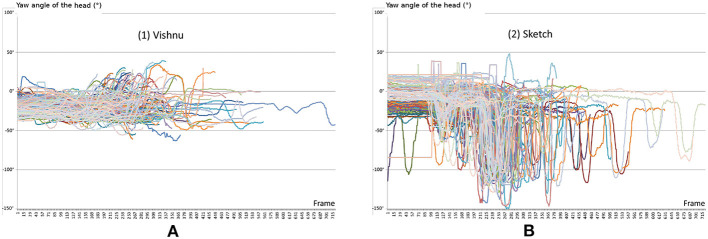
**(A)** Yaw angle of the head for all iterations and all subjects for the Vishnu mode. **(B)** Yaw angle of the head for all iterations and all subjects for the Sketch mode. This illustrates the advantage of the fully collocated setup (mode 1) with a smaller range of 103 vs. 195° for mode (2). In sketch mode (2), we can identify of the movements of the subject's head between the desktop screen and the real targets' space.

As illustrated in Figure 6, the desktop screen mode (2) generates much more head movement than technique (1), giving a yaw angle range of 195 vs. 103°, respectively. This result and our validated hypothesis (especially in a complex scenario) prove that our *Vishnu* paradigm is able to perform better than other techniques in terms of task completion time, while increasing the levels of naturalness and collaborator awareness.

### 4.3. Navigation Guidance

We propose a novel interaction technique based on a MR system in which a remote expert using a VR application guides an agent using an AR application. The agent must perform a physical task that is guided by virtual arms collocated with his or her shoulders, controlled by the remote expert. In our previous pilot study, we simplified our scenario by using a seated operator. More realistic cases are more complex. Indeed, before performing the actual arm guidance, the remote expert must reach the correct location in the virtual world while showing it to the agent in the augmented real world. To perform this initial location guidance, we propose to extend the GoGo technique initially developed for single user selection and manipulation tasks in VR (Poupyrev et al., 1996). We add the arms representation as described by Fraser et al. (1999) and remove the non-linear scaling, instead controlling it directly via the expert's interactions.

#### 4.3.1. Stretchable Guiding Arms

The stretchable guiding arms technique was developed to overcome the limitations found in the literature in the specific context where the expert and the agent share a fully collocated setup (Le Chénéchal et al., 2015). The final goal of the system is to guide the agent in performing a physical task with the use of virtual arms controlled by the remote expert. In this setting, the first step consists of the collocation of viewpoints. The expert must then indicate which object to move, and therefore often needs to move the agent's viewpoint forward, in order to grasp the virtual cloned object. The existing technique is based on a viewing frustum that represents both the location of the expert's head and his or her field of view, in order to improve awareness between collaborators; this is combined with directional arrows that show the path to reach this same viewpoint, as illustrated in Figure 8. However, the first users who tested our system complained of eye strain due to the proximity of visual cues, including the remote expert's frustum. This also increases the cognitive load of the agent, who needs to follow the guidance of the virtual arms and the location guidance simultaneously, using different visual cues^1^. To improve this situation, we take advantage of the virtual arms, which were initially designed for gesture-based guiding purposes only. These can also be used for location guidance in a non-intrusive way, unlike other methods. Figure 10 illustrates the principle of the proposed stretchable guiding arms technique. When the expert chooses to switch to stretching mode, then from the agent's viewpoint this forward motion makes the virtual arms start to scale up, in order to keep the virtual hands collocated with the expert's, while the virtual shoulders are fixed to the agent's shoulders. From the expert's viewpoint, the virtual arms do not scale up, and remain collocated with his or her shoulders.

**Figure 7:**
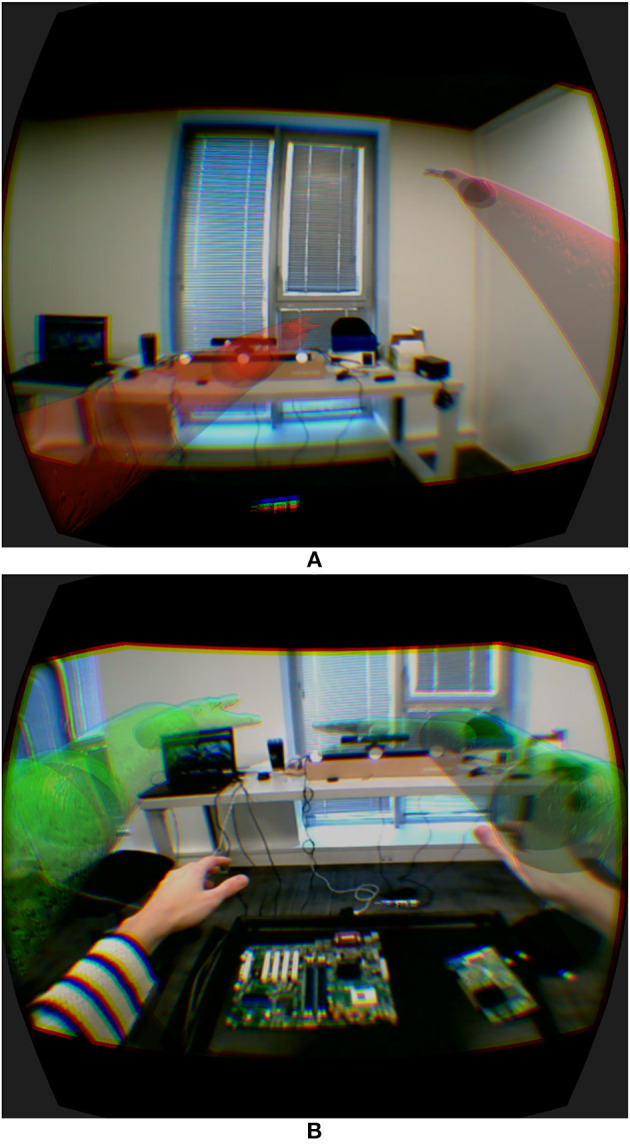
Stretchable guiding arms, controlled from the remote expert's location, from the agent's viewpoint. **(A)** In the top image, the arms are extended, and are shown in red because the guide is ahead of the agent. **(B)** In the bottom image, the agent has reached the guide's location and the guiding arms return to their initial length and are shown in green.

The main advantage of this technique is the naturalness of the guidance. The agent can directly perceive the forward motion of the remote expert, thanks to the scaling up of the virtual arms. Then, to follow the expert, the agent simply moves his or her viewpoint in order to return the virtual arms to their initial length. In addition to the scaling cue, the color of the independent virtual arms changes smoothly from green to red, according to their scale. The arms return to green when the scale is close to its initial value, meaning that the agent has reached the expert's viewpoint (Figure 7). If the agent overshoots the expert's location, the virtual arms keep their initial length and color but bend to inform the agent to move backward to the correct position.

In parallel, the expert can use a virtual camera with a different viewpoint of the VE in order to get visual feedback on the agent following. This camera is displayed in a corner of the expert's viewport and is enabled when the stretchable guiding arms technique is activated. The remote expert can switch between different available viewpoints: a side or front view based on his/her head location, or the viewpoint of the agent, which is slightly behind the expert's when performing forward motion guidance. A change in color of the virtual arms from green to red is used to inform the remote expert explicitly about his/her distance from the guided agent.

#### 4.3.2. Pilot User Study

**Figure 8:**
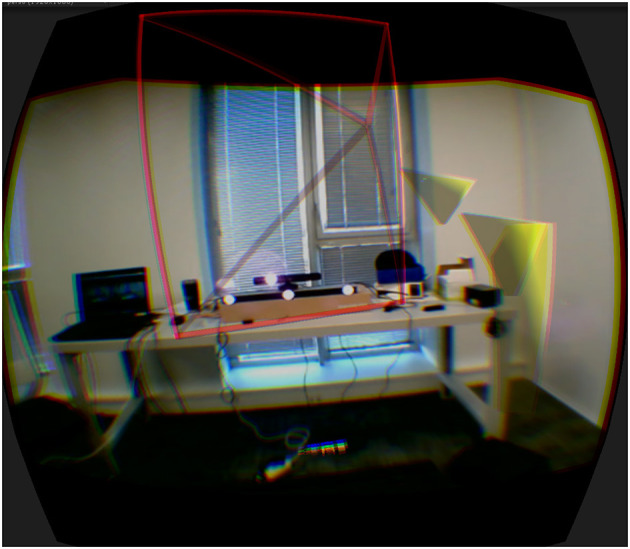
A simple guiding technique based on the viewing frustum of the remote expert and a continuous path made up of directional arrows. This aims to mimic the guiding arms technique, especially in the case where the expert's frustum is not visible from the current operator's viewpoint.

We compared the performance and subjective feelings of (C1) our technique (Figure 7) vs. (C2) the frustum with a 3D directional arrows-based approach (Figure 8), see also additional Supplementary Video 1 for a better understanding of the situation. In order to evaluate the agent's performance in the process of reaching a location, we tried to remove bias generated by the expert's interactions. We therefore recorded the interactions of a real expert in the pre-process phase in order to replay these for the experiment, thus removing any possible bias due to changes in behavior. We postulate that the expert's interactions are not strongly affected by those of the agent. The expert must reach a location to grab a virtual object, and must therefore move forward to achieve this goal, assuming that the agent is following.

##### 4.3.2.1. Measurements and hypotheses

We recorded three objective measures: the completion time (to hit a target at around 0.15 m), the mean distance between the replayed expert and the subject, and the final precision (triggered by the subject) in terms of location and orientation. Subjects filled in a final subjective questionnaire (using a seven-point Likert-scale) to express their impressions of the naturalness of the system, their perceived cognitive load and eye-strain, amongst other considerations. We put forward the following hypotheses:

- H1: Completion time, mean distance and precision are equivalent under conditions C1 and C2;
- H2: C1 is more comfortable to use than C2.

##### 4.3.2.2. Protocol

**Figure 9:**
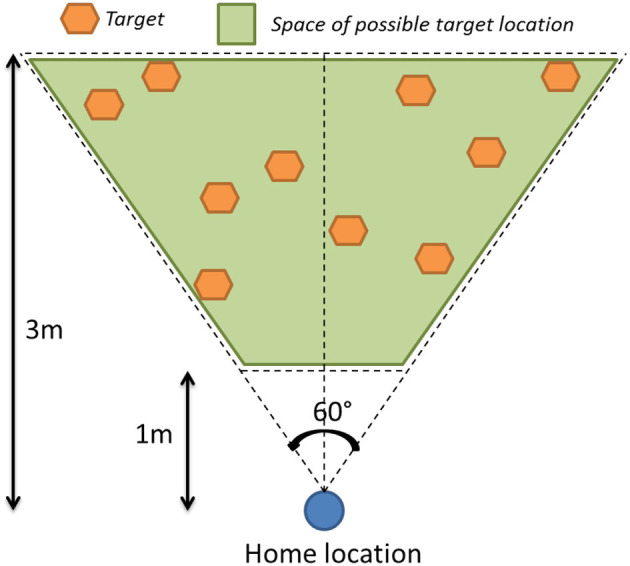
Example of 10 targets defined for one condition.

**Figure 10:**
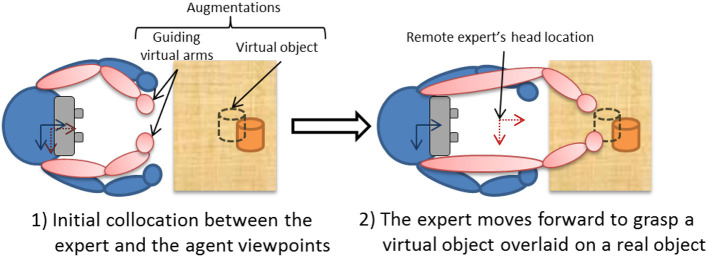
Illustration of the principle of the stretchable guiding arms technique. The agent's side is shown in blue. From the expert's viewpoint (shown in red), the virtual arms do not scale and also remain collocated with his or her shoulders.

We designed our experiment in a counterbalanced way, using a set of 20 pre-defined targets (10 per condition) within a range of 1–3 m from the home location, and a horizontal angle range from −30 to 30° (cf. Figure 9). The altitude was defined around a mean value of 1.5 m, with a range of ±0.3 m. Each participant passed both conditions twice alternately, with a different set of 2 × 5 virtual targets per condition. These targets were not displayed during the replay process, as the subjects were asked to reach them following only the recorded guide. We simulated the collocation of initial viewpoints, as illustrated in step 1 of the process described in Figure 10, with a re-targeting of the replayed data according to the subject's height and exact home location.

##### 4.3.2.3. Results and discussion

The detailed results can be found in Le Chénéchal et al. (2015) here, we only recall that the delay in hitting the target between the guide and the subject was shorter with the guiding arms than with the frustum, but that frustum-based guiding was significantly more accurate than the guiding arms for both location and orientation.

Hence, H1 is rejected. We found that the guiding arms were more effective in closely following the remote guide, while the frustum-based guiding was more accurate in terms of final precision. This degradation of accuracy when using the guiding arms can be explained by the complexity of the DOFs of the human body, which generate variations between the relative pose of the agent's head and the expert's hands. Thus, the coupling of the guiding arms with an alternative guiding technique for final positioning should be studied, with the aim of improving accuracy without decreasing the naturalness of the guidance. The qualitative results showed that H2 was validated, as there was better visual comfort using the guiding arms. Moreover, subjects preferred the guiding arms in terms of their originality and the presence of the guide. For the other criteria (efficiency, naturalness and cognitive load), the results were similar for both conditions.

#### 4.3.3. Extension

**Figure 11:**
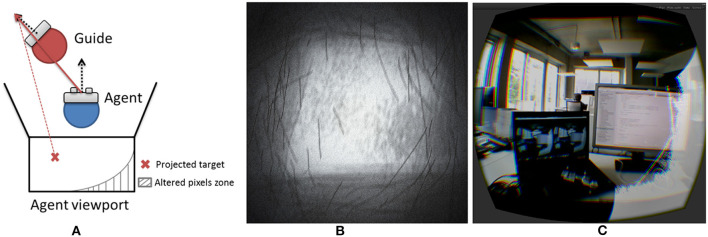
**(A)** Principle of the guiding extension to improve final precision (top view); **(B)** noisy reticule texture used to alter pixels within the guiding shader; **(C)** illustration of the guiding shader performing as an extension of the stretchable guiding arms. The white zone is the alteration zone, indicating here that the guide is looking to the left and is a little higher than the current follower's viewpoint.

Since the results were better for C2 in terms of the final precision of position and orientation, we introduced an additional guiding cue with the C1 technique. This extension was enabled as soon as the agent was close enough to the guide, and handled both position and orientation guidance. Thus, it provided a final six-DoF guiding cue in order to improve final positioning. This extension was based on an alteration of the final rendering in order to guide the agent in the right direction while taking into account the current position. Figure 11A illustrates the way in which the pixels are computed, altering according to both users' positions and orientations. This was implemented using CG fragment shader and a reticule texture (Figure 11B) was used to alter pixels. Figure 11C illustrates its action from the agent's field of view.

This additional guiding cue needs to be formally evaluated, but initial testing indicates that this is a good way to improve our stretchable guiding arms technique in terms of its final precision. It generates a visual discomfort in the follower's field of view that tends to reorient the follower toward the guide's point of view. At the same time, its implementation is not disturbing, as it acts only in the peripheral vision of the user, while avoiding the use of the guide's frustum, which generates uncomfortable virtual interpenetration between the follower's point of view and the guide's virtual frustum.

## 5. Asymmetrical Approach and Navigation Guidance

Existing remote guidance systems for collaborative maintenance are mainly based on streaming 2D video to the expert, as captured by the agent's camera. The agent is then helped via a hand-worn AR interface.

However in AR, from the operator's side, several perception issues are introduced due to unhandled occlusions between real and virtual objects that create depth cue conflicts, causing difficulties in properly perceiving the relative depth. In our setting, we handle this feature with the use of a depth camera and the 3D reconstruction of the real environment. This enables streaming of this 3D reconstruction to the expert, showing the maintenance space in 3D. The expert can move his or her own PoV around the remote reconstructed workspace. Lastly, several interaction tools are provided to the expert, who can:

- point out items to the agent using a 3D ray;
- add/remove virtual arrows in the shared VE in order to add fix virtual pointers into the shared VE;
- segment parts of the reconstruction to create movable virtual objects;
- guide the agent using the navigation technique described in section 5.2.

**Figure 12:**
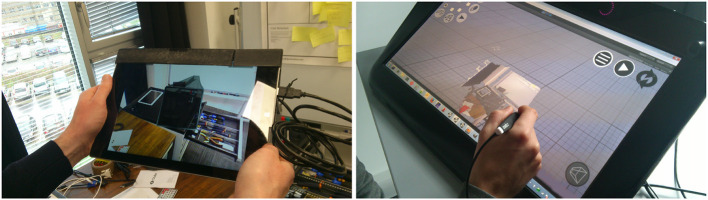
Illustration of the asymmetrical (HWD and WIM) prototype. The agent on the left is guided by the remote expert on the right.

Figure 12 illustrates our prototype in practice.

### 5.1. Implementation

**Figure 13:**
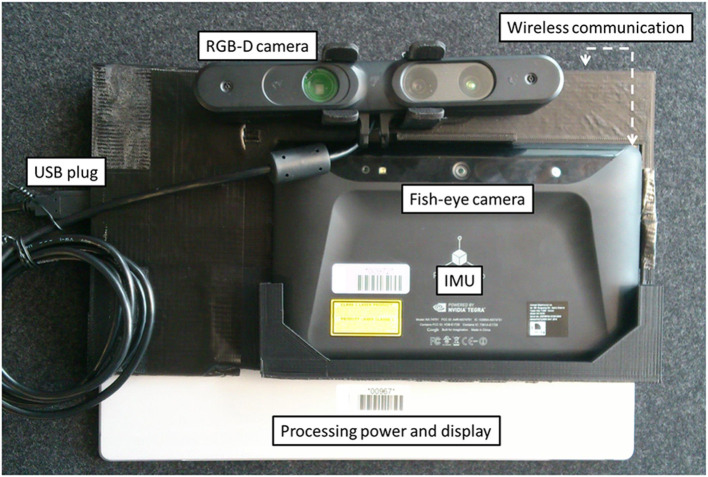
The back of our AR tablet prototype. The Google Tango tablet uses sensor fusion with a fish eye camera and IMU to provide a SLAM-based six-DoF tracking. The RGB-D camera is a Asus Xtion powered with USB. These two devices are mounted using a 3D printed support on a Microsoft Surface Pro 3 (Intel CoreI7 with HD Graphics 5000 chipset) which provides processing resources as well as a large display.

The developed AR device prototype is a wireless tablet PC (Microsoft Surface Pro 3) coupled with a Google Tango device, which was used to compute six DoFs and was self-positioning in real space (using inside-out tracking based on SLAM, with a wide field of view fused with IMU data) and was equipped with a high-resolution USB-powered depth camera (Asus Xtion Pro). This setup handles real-time 3D reconstruction from a 640 × 480 depth map provided by the depth camera as well as marker-free tracking. Figure 13 illustrates this setup. The setup needs a registration phase to define the rigid transformation between the fish-eye tracking camera and the RGB camera used for AR overlay. This is achieved using an OpenCV camera calibration method based on a chessboard image captured by both cameras at the same time. The rigid transformation is the difference between the two computed poses according to the chessboard. Notice that although the Tango tracking tablet is also equipped with a depth camera, this tablet does not have enough processing power to perform real-time 3D reconstruction at high resolution (the depth camera produced a sparse depth map of #10,000 points, i.e., resolution #100 × 100). The current expert's setup is a zSpace that provides head-tracked stereoscopic display and a six-DoF stylus for interaction.

### 5.2. Navigation Guiding

The main advantage for the expert with this setting is the ability to dynamically discover the remote workspace based on his/her own guidance. Thus, the expert can build his/her own awareness of the remote workspace and operator's activity using this dynamic 3D reconstruction for discovery feature.

**Figure 14:**
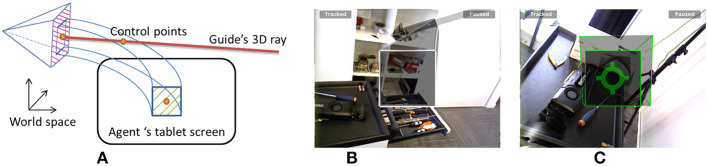
Squared guiding arrow principle controlled by the expert's 3D ray in dynamic mode. **(A)** Scheme of the control principle, constrained by three control points; **(B)** the agent's display showing a PoV to reach dynamically controlled by the expert's 3D ray (seen on the top right of the screenshot); **(C)** the agent has reached the pointed spot, and the base and the end squares of the arrow are aligned and almost superposed. Notice that the green reticule in the center of the base square shows a potential additional cue that used in combination with the proposed technique. This acts as a flickering proximity sensor to improve the agent's fine positioning. We did not enable this additional feature for the pilot user study in order to evaluate performances of our proposed technique removing other helping biases.

In our prototype, the occlusion handling in the operator's AR display is useful to provide a good perception of depth for virtual objects integrated into the real world; however, this may also hide some of the visual guiding cues controlled by the remote expert. Furthermore, if the expert wants the agent to go to a location to the side of or behind the agent's field of view, it can be difficult to perform navigation guidance efficiently. Thus, we propose a navigation technique that gives visual continuity between the agent's HWD and the target spot. It is composed of a 3D arrow, made up of a spline controlled by three control points, with six DoFs (Figure 14).

On the agent's side, the base of the squared arrow (i.e., the base control point) is fixed relative to the HWD AR display. The agent must move this, in order to align this square with the square formed by the end of the arrow. Once this is done, a proximity sensor is displayed and flashes according to the distance to the final location (using position and orientation differences). The final location is correct as soon as both squares (i.e., the base and the final squares) are superposed.

The expert can guide the agent using two modes. The 3D ray means that the static mode can be used to define the point of interest as well as the orientation of the desired agent's viewpoint. The expert then specifies the radius of a spherical volume centered on this point of interest, which will represent the bounding volume that the agent should view to operate. Last, the system computes the locations of the two final control points to place the guiding arrow. When the agent reaches the pointed spot, the camera will contain the defined volume in the agent's viewing frustum. In dynamic mode, a 3D ray is also used to continuously define the position and direction of the desired viewpoint for the agent. Here, the two final control points are computed dynamically when the expert moves the 3D ray. The first control point is at the ray extremity, and the second is at a variable distance from the ray extremity, depending on the desired spline curvature. In dynamic mode, the expert continuously controls the end of the squared guiding arrow and can refine the guidance while discovering the remote workspace.

#### 5.2.1. Experiment

We conducted an experiment on the agent's side in order to evaluate the effect of the visual continuity introduced with our navigation technique.

##### 5.2.1.1. Protocol and hypotheses

We compared the performance in terms of reaching a statically defined target using our technique (mode 1) vs. a simple guiding technique based on a compass and a 3D cylindrical pointer (mode 2) that was based on commonly used techniques. The compass was inspired by Nguyen et al. (2013) and the cylindrical pointer by Tonnis et al. (2005). In mode 2, the compass always points in the direction of the target. This is useful in indicating its location when it is hidden by real furniture or walls, for instance. Once the target is within the field of view of the agent's camera, the cylindrical pointer is used to indicate the exact PoV to reach in order to achieve the desired perception of the target.

**Figure 15:**
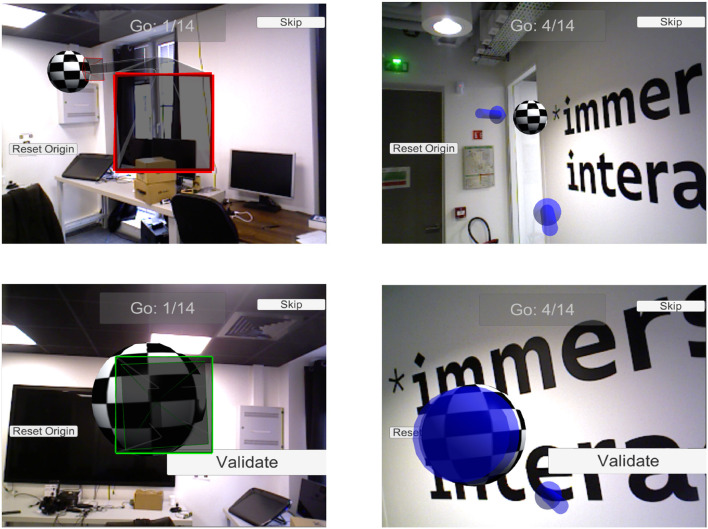
The two guiding techniques compared in this study. The squared guiding pointer is shown on the left, and the pointer with compass on the right (the compass is the small cylindrical pointer at the bottom of the viewport). The checkered sphere is the target on which subjects must reach a specific PoV. In the lower images, the user reaches the designated target and the PoV is sufficiently aligned with the desired PoV to validate the final precision.

We chose a static guiding condition because it allowed us to remove any potential bias that could have been generated by an expert's interactions dynamically defining a target to reach. In order to follow the superposition of squares principle used in mode 1, the correct location in mode 2 is also reached when the bottom circle is superposed with the larger ring of the cylindrical pointer. Figure 15 illustrates the two techniques compared here and the alignment conditions.

**Figure 16:**
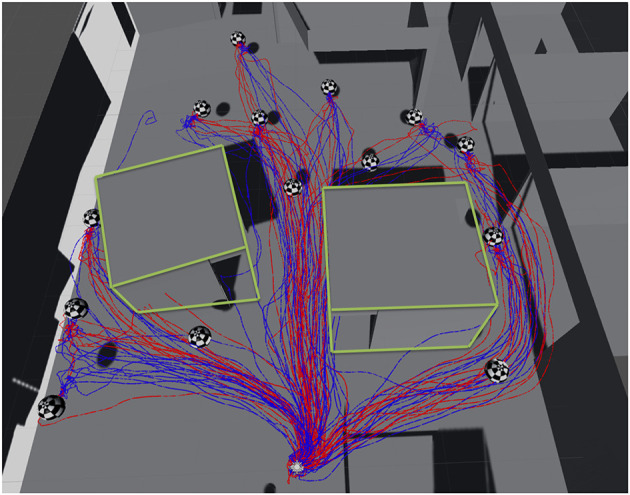
The location of targets on the floor, and the paths of the participants, recorded during the experiment (blue: mode 1; red: mode 2). The green outlines represent the walls of two rooms present in the space.

Initially, the subjects tested both guiding modes twice, and then performed five iterations of reaching the target for each mode (Figure 16). After each iteration, subjects returned to the original position on the floor before starting the next. The mode order was counter-balanced between all the participants.

We recorded the completion time taken for the subjects to reach a threshold close to the target location (in terms of position and orientation). They could then take their time to achieve a final position as closely as possible to the perfect location indicated by the current guiding metaphor (i.e., perfect superposition of squares in mode 1 or circles in mode 2).

Our hypotheses for this stage were as follows:

- H1: Completion time is lower using mode 1 than mode 2, since the continuity aspect reduces the ambiguity of the guiding metaphor compared to a compass that points into the void.
- H2: The precision, especially in terms of orientation, is better for mode 1 than mode 2 because the superposition of squares allows better 3D orientation perception than circles, which do not contain helpful angular visual cues.

##### 5.2.1.2. Results

**Figure 17:**
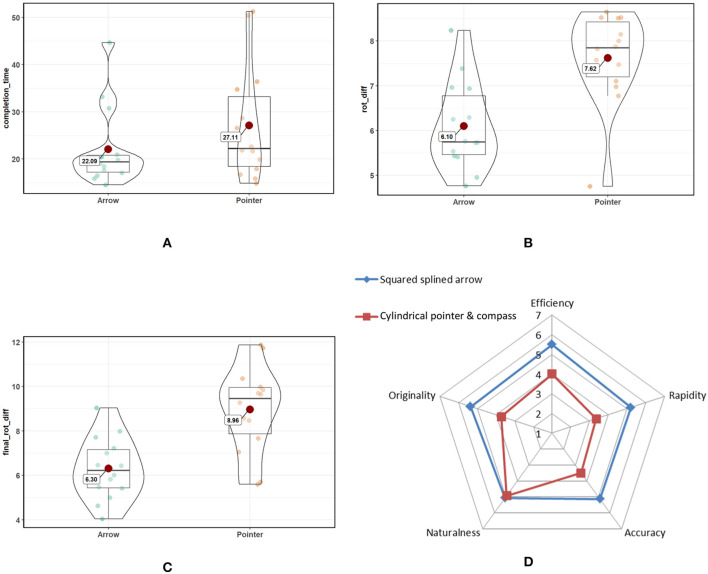
**(A–C)** Boxplots of collected quantitative results; **(D)** summary of qualitative results.

We collected data from 14 subjects aged between 22 and 54 (*mean* = 27.9, *std* = 7.9). The illumination conditions sometimes disturbed the vision-based tracking performed by the Tango device, and we discarded these iterations (~ 15%) from the analyzed results. Figures 17A–C illustrate the results of ANOVA tests. The completion time (in seconds) was significantly lower in mode 1 (*M* = 22.09, *std* = 8.39) than in mode 2 *(M* = 27.11, *std* = 11.99*):* *F*_(1, 13)_ = 3.76, *p* = 0.07, *small effect size =* 0.22. In terms of positional precision, we found no significant differences between the two modes. However, orientation precision was better for mode 1 than mode 2. In the approach phase (i.e., the time in which the system validates the agent having reached the target), subjects reached targets with a significant better angular precision in mode 1 (*M* = 6.10°, *std* = 0.97) than in mode 2 (*M* = 7.62°, *std* = 1.02): *F*_(1, 13)_ = 27.74, *p* < 10^−3^, effect size = 0.68. In terms of the final angular precision (without taking into account the roll angle), mode 1 (*M* = 6.30°, *std* = 1.38) was significantly more accurate than mode 2 *(M* = 8.96°, *std* = 1.93*):* *F*_(1, 13)_ = 26.35, *p* < 10^−3^, *effect size* = 0.67.

Lastly, the qualitative results show that subjects preferred mode 1 over mode 2 for every criterion (Figure 17D). An exact Wilcoxon-Mann-Whitney test shows significant differences (*p* < 0.01) for each criterion except naturalness.

##### 5.2.1.3. Discussion

Both of our hypotheses were confirmed. First, H1 proves that our technique, which uses a continued squared arrow driven by a spline, is easier to follow than a simple direction shown by a compass. This means that the remote target is reached faster, thus improving the overall process of remote guiding. Secondly, H2 demonstrates that the use of square shapes is much more efficient in a 3D space than circle-based shapes, since squares convey angular information, unlike circles. These angular visual cues are very important in providing an efficient way to reach specific orientations within a 3D space. Obviously, the roll angle cannot be defined using the superposition of circles. In our approach, this roll angle is handled by the third control point attached to the agent's display and the continued spline that defines the whole guiding arrow. Pitch and yaw are also easier to perceive, since the deformation of a square seems to carry more 3D information, thus making it easier to perceive than the deformation of a circle, which creates a slightly ellipsoid shape.

## 6. Conclusion

In this paper, we develop ideas around the use of MR technologies to improve remote collaboration. In particular, we apply these ideas to maintenance in order to allow a remote expert to help an agent perform a procedure. We distinguish between two phases in this scenario. First, the remote expert must be able to analyse the agent's workspace in order to diagnosis the procedure to perform. At the same time, the expert must guide the operator to the correct location. Thus, we propose a guiding technique for remote navigation. In the second stage, physical manipulation can begin, and we propose guiding techniques for selection and manipulation tasks in this phase.

In this context, we investigated two kinds of systems. The first is a symmetric approach that aims to free the hands of the operator while improving the expert's interactions capabilities and enhancing the operator's perception of the guiding visual cues. Here, the remote expert is immersed into the operator's workspace at a 1:1 scale. We provide the expert with two virtual arms in order to interact with virtual objects. The main idea of our proposal is to collocate the expert with the operator's point of view. The virtual arms, positioned from the operator's shoulders, are used as natural guiding cues that show which objects to grab and how to move them. The second system investigated here is an asymmetrical one, inspired by existing systems based on HWDs with remote camera streaming. We improved this approach using 3D capturing of the operator's workspace in order to provide an enhanced AR view for the operator, and better perception, including depth perception, for the expert. This provides 3D interactions for the expert using 3D visual guiding cues that are perfectly integrated into the operator's real world, in order to help in achieving the procedure.

We ran several pilot user studies to evaluate the efficiency and acceptance of our prototypes. The results showed good performance for our systems compared to existing approaches. When navigating, especially within a building with corridors and complex room shapes, our approaches ensure continuity between the operator and the target spot, allowing the operator to be constantly aware of the expert's guidance. When actually performing the physical manipulation in order to achieve the maintenance procedure, our user evaluation shows that participants perform better with our symmetrical, fully collocated setting when dealing with complex layouts, such as computer racks or electrical panels. However, we identified several drawbacks in terms of the precision of the navigation guidance in this setting, due to the use of a less accurate but more natural and less intrusive visual guiding cue. We therefore propose that an extension aiming to improve this aspect should be evaluated in future work. Further experiments should be run in order to formally evaluate the performances of both settings in realistic scenarios.

### 6.1. Perspectives

In this work, we separated our two proposals into two different systems: symmetrical and asymmetrical. Our findings show that based on the situation and the phase of the procedure, the expert may need to freely navigate into the remote operator's workspace, possibly at a different scale, or may need to share the operator's point of view to better understand the situation. Thus, both settings should be merged into a unified system that can handle both of these situations. We could even imagine a system that would combine both types of display, i.e., hand-worn and head-worn, and allow the operator to switch from one to another, or even to use both at the same time to provide additional capabilities for the expert to offer guidance.

## Data Availability Statement

All datasets generated for this study are included in the manuscript/Supplementary Files.

## Ethics Statement

All subjects enrolled in the pilot user studies presented here provided verbal informed consent. Approval was not required as per institutional guidelines and national legislation in the context of these studies.

## Author Contributions

This work results from the Ph.D. thesis of ML. This Ph.D. thesis was directed by BA and co-supervised by TD, VG, and JR. So this paper is a synthesis of all the contributions that these five people have proposed during the Ph.D. thesis to enhance collaboration in Mixed Reality.

### Conflict of Interest

The authors declare that the research was conducted in the absence of any commercial or financial relationships that could be construed as a potential conflict of interest.

## Footnotes

^1^Notice that we do not consider using an avatar to represent the expert; this would require full-body tracking since in our scenarios, other parts of the expert's body are not relevant and are not used to help the remote agent. This would therefore result in increasing the agent's cognitive load, which involves following multiple visual cues, while not improving collaboration performance.
